# The Effect of Feeding Chicken and Geese Broilers with Different Cereals on the Fatty Acids Profile in Meat

**DOI:** 10.3390/foods10112879

**Published:** 2021-11-21

**Authors:** Piotr Janiszewski, Dariusz Lisiak, Karol Borzuta, Eugenia Grześkowiak, Tomasz Schwarz, Urszula Siekierko, Krzysztof Andres, Sylwester Świątkiewicz

**Affiliations:** 1 Department of Meat and Fat Technology, Prof. Wacław Dąbrowski Institute of Agricultural and Food Biotechnology—State Research Institute, Głogowska 239, 60-111 Poznań, Poland; piotr.janiszewski@ibprs.pl (P.J.); karol.borzuta@ibprs.pl (K.B.); eugenia.grzeskowiak@ibprs.pl (E.G.); urszula.siekierko@ibprs.pl (U.S.); 2 Faculty of Animal Science, University of Agriculture Kraków, Al. Mickiewicza 21, 31-120 Kraków, Poland; tomasz.schwarz@urk.edu.pl (T.S.); krzysztof.andres@urk.edu.pl (K.A.); 3 Department of Nutrition Physiology, National Research Institute of Animal Production, 32-083 Balice, Poland; s.swiatkiewicz@izoo.krakow.pl

**Keywords:** hybrid rye, geese and chicken broilers, fatty acids profile

## Abstract

The research was conducted on the effect of bird broilers fed with different hybrid rye doses on the fatty acids profile in muscle. The first experiment was performed on 3 geese broilers groups fed with hybrid rye, oats or hybrid rye and oats mix in proportion 1:1. No effect of the hybrid rye feeding of geese on the SFA level in meat was observed, but the MUFA level was significantly higher and PUFA level and n-6/n-3 PUFA ratio were significantly lower than in geese fed with oats. The second experiment was performed on 3 chicken broiler groups fed with mix of corn, wheat, soybean meal and rapeseed oil (control group), and fed with an addition of 10% or 20% hybrid rye in diet (experimental groups). No effect of hybrid rye feeding of chicken broilers on the meat quality and SFA level was observed. However, the MUFA level was higher and the PUFA level and n-6/n-3 PUFA ratio were lower in meat of chicken broilers fed with hybrid rye. In conclusion hybrid rye is a healthy ingredient in the diet of studied birds and may be used up to 20% in chicken broilers and 50% in the diets of geese. A 100% hybrid rye in geese diet caused lower final body weight.

## 1. Introduction

The feeding level and chemical content of feed mixtures affect animals’ growth and hormone’s metabolism, as well as the quantity and quality of fat deposited during the daily gain [1]. One of the basic indicators of feed quality is the fatty acids content. It is especially important in mono-gastric animals whose deposited fat may be modified by changing the fatty acids profile in the feed. The natural digestion of lipids is crucial for fatty acids transfer from the feed to the product of animal origin [2]. In ruminants, microorganisms have a significant influence on fatty acid content in food, leaving the rumen and absorbed by the small intestine whereas, in mono-gastric animals such as poultry species, the fatty acids are absorbed unchanged by the small intestine into fat tissue. The source of lipids in feed has a direct and predictable effect on the fatty acids profile in poultry and consequently on their content in meat and meat products [3].

The fatty acid profile in food plays an important role in human health. The higher share of unsaturated fatty acids in the diet reduces the risk of heart diseases, diminishes the risk of some types of cancer, asthma, and diabetes [2]. There is a hypothesis that it is not the quantity of meat but the quantity of fat and its composition that is carcinogenic [4]. The research of Schulz et al. [5] showed the relation between consuming a lot of processed meats, fish, butter, and other fats of animal origin, as well as margarine accompanied by small amounts of bread and fruit juices, and a higher risk of breast cancer in women. The amount of fat in the diet and the fatty acids profile did not have any influence on the risk of prostate cancer in men [6]. The research proved to have a random/selective effect of the quantity and quality of consumed fat on the risk of developing cancer. The MUFA and PUFA, including n-3 omega acids, play a key role in cancer prevention, but their share in meat is low [7].

Feed for poultry produced in Poland is based on cereals with high C 18:2 n-6 acid content and low C 18:3 n-3 acid content. However, the fatty acids profile varies significantly depending on the cereal type. For example, the C 18:2 n-6 and C 18:3 n-3 ratio, according to research, was for rye varieties 6.0–7.2:1, for the hybrid rye Fernando variety it was 6.4:1, for oats it was 12.4–22.7:1, for winter wheat it was 10.9:1, for winter barley it was 9.7:1, and for corn it was 33.7–43.8:1 [8]. In recent years, very intensive work has been conducted on rye genetics. The work was concentrated on developing crops with reduced amounts of anti-nutritional substances [9,10,11]. New hybrid rye varieties produced a higher yield and had reduced the level of the anti-nutritional substances [12]. Furthermore, the hybrids grow on poor and average soil, tolerate soil acidification well, and are frost-resistant and cheaper than other cereals [9]. As a result, the profitability of using hybrid rye in animal feeding is higher compared to other cereals.

Despite the growing interest in feeding poultry with hybrid rye, the knowledge of the effect of this feed on the fatty acids profile has not been widely investigated. Therefore, this research aimed to examine the effect of the hybrid rye variety on chicken broilers and geese fatty acids profile in poultry breast meat.

## 2. Material and Methods

### 2.1. Diets and Animals

Two separate experiments were performed on the influence of the hybrid rye Brasetto variety in feed on the fatty acids content in geese and chicken broilers meat. A different experimental pattern was applied for each poultry species.

#### 2.1.1. Geese

An experiment was performed on young geese of the Polish Zatorska rare breed, a meat type that is a part of the genetic resource protection program. The experimental plan is presented in Table 1.

There were 300 goslings in the flock (150 young ganders and 150 young geese) that were moved after hatching and sexing, to the rearing building with 7 birds per sq m in the bedding system on the straw, with natural light, and with a starting temperature of 28 °C lowered to 22 °C in the third week of life. The birds had access to feed and water. They were fed with compound feed no. 1—the feed content is presented in Table 2. Starting from the 3rd week, the goslings were kept on straw bedding with 3.6/sq m stocking density in a building with windows, and with free access to pasture for at least 8 h a day. The temperature in the rearing building was 20–22 °C and relative humidity was 65–70%. Between 4 to 14 weeks, the birds were fed with compound feed no. 2 (Table 2). After 98 days of life, the birds were divided into 3 groups of 100 each (50 males and 50 females in each of the groups). They were fed respectively with the hybrid rye Brasetto variety (group A), oats (group B), and mixture 1:1 of the hybrid rye Brasetto variety with oats (group C). The chemical composition of hybrid rye and oats is presented in Table 3. The fattening was performed in boxes with 1 bird/sq m stocking density on the straw bedding with 8 h access to outside straw paddocks. The temperature in the boxes was 12–18 °C and the relative humidity was 65–70%. The fattening lasted until the birds were 17 weeks old, and after 10 h of fasting, the birds were slaughtered in the commercial slaughtering plant. From each group, 24 carcasses were randomly chosen for laboratory analysis (sex ratio as 1:1).

#### 2.1.2. Chicken Broilers

A total of 72 1-day-old, male Rose 308 chickens were used. These were obtained from a commercial hatchery and had an average initial weight of 41 g. The birds were housed in wire-flooded cages, in an environmentally controlled room in the poultry house at the Experimental Station of the National Research Institute of Animal Production in Balice, Poland. During the study, the temperature in the experimental facility was maintained from 32 °C at 1 day of age to 21 °C at 21 days of age, and later, relative humidity cycled from 50 to 60%, air exchange was 1 m^3^/1 kg of BWG/1 h and the concentrations of CO_2_ and NH_3_ were maintained below 2000 and 20 ppm, respectively. The chickens were weighed with BD3T electronic scales with an accuracy of 0.1 g (AXIS, Gdańsk, Poland) and randomly assigned to 1 of 3 treatments, each comprising 3 replicate cages, with 8 birds per cage (with 7800 cm^2^ total floor space in the cage). From 1 to 42 days of age, all the chickens were provided with water and feed ad libitum.

All the birds were reared up to 42 days of age and fed with crumbled starter (1 to 21 days) and pelleted grower-finisher (22 to 42 days) isonitrogenous and isoenergetic diets (Table 4), which were formulated to meet or exceed the nutrient requirements of broilers [13,14]. Rye grain was ground using a 5-mm sieve size. Three dietary levels of ground rye of Brasetto variety (0%, 10%, 20%) were evaluated in the forage for broiler groups A, B, C, respectively.

Broilers were slaughtered at 42 days of age in the commercial slaughterhouse conditions.

### 2.2. Sampling

The geese carcasses after slaughter were chilled in the day-night cycle at 4 ºC. The carcasses for tests were randomly chosen in the scheme of 12 male and 12 female carcasses from each of the 3 experimental groups (24 samples per group). Next, 48 h after slaughter, the breast muscle was cut out (*M. pectorialis major*) for lab tests.

### 2.3. Lipids Analysis

The fatty acids profile was determined in the intramuscular fat from raw breast muscle. The methyl esters of the fatty acids samples were prepared according to the PN-EN ISO 12966-2:2017-05 method [15]. The fatty acids composition was determined by gas chromatography using a Hewlett Packard HP 6890 device (Agilent Technologies, Santa Clara, CA, USA), equipped with a flame-ionic detector and a high polarized column with a BPX 70 phase. The column was 60 mm long, a layer thickness—0.25 μm, whereas its internal diameter was 0.22 mm. The analyses were performed in the programmed temperature and time. Individual fatty acids were identified by comparison of retention times to those of a standard FAME mixture (Supelco 37 Component FAME Mix and C18 FAME Isomers, Sigma-Aldrich, Schnelldorf, Germany) and expressed as a relative proportion of all of the FA in the sample.

### 2.4. Analytical Determinations

The physical traits of the muscle were analyzed 24 h post-mortem as well as its sensory traits. The pH of the meat and the electrical conductivity were measured using respectively: a Sydel pH-meter with dagger electrode, 15′ and 24 h after slaughter. Meat colour was determined on the muscle cross section using a Minolta Chroma Metters CR 400 device produced by Konica Minolta and parameters of the colour were in L*a*b* scale (light source D65, observer 2°, head slot 8 mm, calibration on the white standard: L* 97.83; a* 0.45; b* 1.88).

Water holding capacity (WHC) was measured with the Grau and Hamm method [16] modified by Pohja and Niinivaara. The sensory estimation of the cooked muscle was performed in a 5-point scale, including smell, juiciness, tenderness, and flavor. The sensory test was done by 4 scientists (experts) from the Prof. Wacław Dąbrowski Institute of Agricultural and Food Biotechnology, trained and tested for sensory sensitiveness. The assessment was made in daylight at room temperature. In a 5-point scale: smell, juiciness, tenderness and palatability, were evaluated. The following grading scale was used: smell: 1—very unacceptable; 5—very acceptable; juiciness 1—very dry; 5—very juicy; tenderness; 1—very tough; 5—very tender; palatability; 1—very unacceptable; 5—very acceptable.

### 2.5. Statistical Analysis

The obtained data were subjected to the analysis of variance (ANOVA) using Statistica 6.0 software [17]. Tukey’s test of multiple comparisons was used to determine significant differences between mean values (*p* < 0.05). The results are presented as average values and standard error of the mean (SEM).

### 2.6. Ethics Statement

All experimental procedures performed on live animals followed the EU Directive 2010/63/EU for animal experiments [18] and the Polish law for the care of animals used in research and education. According to Polish law, the ethical approval of research is not formally required if experiments involve only the standard operating procedures typically carried out on a commercial farm. The slaughter of animals aiming to obtain tissues for laboratory analyses is not formally considered, as research procedure and the ethical approval of such action are not required.

## 3. Results

### 3.1. The Effect of Feeding Geese Broilers

The studied groups of geese were obtained different final body weight at seventeen weeks of age (Table 5).

Highest live weight was stated in geese fed with oat, and it was significantly higher than in geese fed with hybrid rye (*p* < 0.05). It was connected with higher energy value and higher fat content of oat in comparison to hybrid rye (Table 3). Oats also contain several times more crude fiber than rye grain, which also affects digestion. The final body weight of geese from group C was not significantly different to the geese from group A nor to group B geese.

The results on the fatty acids profiles determination indicated significant differences in the geese depending on the feed content (Table 5). In the lipids of the breast muscles of geese fed during the last 3 fattening weeks with hybrid rye, a significantly higher MUFA, as well as significantly lower PUFA contents, were reported when compared with the results obtained for the geese fed with oats or rye-oat mixtures. Feeding with oats caused a negative increase in the PUFA n-6 to PUFA n-3 ratio to approximately 17.01, whereas in the groups fed with rye and a rye-oat mixture, this ratio was significantly lower and reached values 13.47 and 13.23, respectively. Meanwhile, the SFA content in the tested muscles did not differ significantly between the examined groups and was approximately 31%. The highest PUFA n-3 content was observed in the meat of geese fed with a hybrid rye-oat mixture, which is crucial in cancer prevention. The total cholesterol level was not significantly different between the groups, and varied from 75.3 to 78.9 mg/100 g of meat.

It is worth noticing that the highest level of C 18:1 oleic acid with a double bond in the cis spatial configuration was reported in the group of geese fed with hybrid rye (43.12%) that was significantly different (*p* < 0.01) from those groups fed with the oat or rye-oat mixture, reaching 39.59% and 41.42% respectively. The oleic acid C 18:1 content in the trans spatial configuration was less favorable in those geese fed with hybrid rye than in the geese fed with oats or a rye-oat mixture (0.32% in group A and 0.27% in groups B and C).

### 3.2. The Effect of Feeding Chicken Broilers

Live body weight before slaughter of chicken broilers at 42 days of the life and the dressing yield were not statistically different between studied groups (Table 6).

The obtained results indicate the significant effect of hybrid rye in the chicken broilers diet on the fatty acids profile in the breast muscle (Table 6). The addition of 20% rye in the forage caused a significant decrease of SFA content, from 25.96% in the control group A to 23.12% in the experimental group C, as well as a significant increase of MUFA from 48.72% to approximately 51.32% being observed. No significant differences in the MUFA levels were detected between 20% and 10% dose of the rye in the feed. The profitable increase of C 18:1 acid in the cis spatial configuration 9 from 41.15% in the control group to approximately 43% was observed in both experimental groups B and C (*p* < 0.01). The trans C 18:1 acid positively decreased from 0.19% in the control group to 0.12% in group B, with 30% rye in the dose (*p* < 0.01). The addition of 10% rye in the dose did not have a significant influence on the C 18:1 trans acid in the meat of the chicken broilers.

The results of the PUFA levels were inconclusive because the lower rye addition in the dose caused their decrease in the tested muscle (*p* < 0.05), and a higher addition did not have such an effect. There were differences between the PUFA n-3 and PUFA n-6 levels that caused a profitable decrease in the n-6/n-3 ratio, with a higher rye share in the dose from 5.8 in the control group to approx. 4.6 in the group fed with a 20% hybrid rye share in the compound feed (*p* < 0.01). The addition of rye also had a significant effect on lowering the cholesterol level in the broilers breast muscle, from 72.5 to approximately 60–64.5 mg/100 g tissue in the experimental groups (*p* < 0.01).

Physicochemical and sensory traits of the chicken breast muscles are presented in Table 7. The studies showed that the addition of hybrid rye in the diet of the birds resulted in higher final muscle acidification (*p* < 0.05) and a reduction in yellowness (*p* < 0.01), as well as an improvement in WHC (*p* < 0.01). However, it had no effect on the sensory characteristics of meat.

## 4. Discussion

Present lifestyles and human eating habits make us prone to delivering surplus amounts of energy to our bodies with all the undesired health consequences. Dieticians recommend reducing fat consumption to the level when energy is delivered by fat in not more than 25–30% of daily intake (FAO/WHO Food Standards, 1984). These recommendations are aimed at delivering optimal amounts of nutrients with the fat dose and zero or limited amounts of undesirable ingredients [19]. Such undesirable ingredients are saturated fatty acids, which are consumed excessively, and thus, make people prone to ischemic heart disease due to their hyper-cholesteroleic effect, i.e., by increasing the total cholesterol level and its LDL fraction, as well as the atherogenic effect (i.e., causing atherosclerosis).

This study showed that geese feeding with the hybrid rye caused the lowering of final body weight in comparison to feeding with oat. However, rye addition in mixture with oat in proportion 1:1 levelled this negative effect on birds’ growth. On the contrary in the chicken broilers feeding rye addition up to 20% in diet had no effect on its growth, because final body weight in all studied groups caused about 3 kg, and there was no significant change between groups. A comparison of final body weight at 40 days of age was stated in the study of McCafferty et al. [20], which caused about 2.8 kg in both investigated groups of chicken broilers fed wheat or corn.

Hybrid rye used in geese feeding did not affect the SFA level in the meat samples, whereas in chicken broilers feeding it caused it to be lower by the 3 pp level compared to the control group, but only when a higher rye dose was added to the diet (i.e., 20%). In chicken broilers fed with a higher rye dose a significantly lower cholesteroleic saturated fatty acids level was confirmed C 12:0; C 14:0 and C 16:0. A significantly lower level of only lauric acid (C 12:0) was also found in the group of geese fed with a rye-oat mixture, compared to the control group and the group fed with rye only.

A similar saturated fatty acids profile was reported in Chech geese by Uhlirova et al. [21]. Geese were fed with a wheat-corn-soy mixture, and the general SFA level in the thigh muscles was approximately 30–32% on average, depending on the breed. The lower SFA level, approximately 27%, was found in the Polish oat geese of the white Kołudzka breed [22,23]. A higher SFA level was reported in the research of the Ukrainian Arzamas geese breed fed with corn, wheat, and oat mixture with 5% soy oil in the dose [24]. In the lipids in the thigh muscles, approximately 29% of SFA acids were detected. A higher SFA level was observed in chicken broilers (34%) fed with a commercial mixture with a share of soy [25].

The atherogenic acids level (arachidic acid C20:0 and behenic acid C22:0) did not depend on hybrid rye feeding in all of the experimental birds. Meanwhile, the MUFA profile was dependent on the rye diet in the geese and chicken broilers. As a result, the rye dose in the diet effect on the higher the MUFA level in chicken groups with 10 and 20% grains (*p* < 0.01), but in the case of geese fed with oats and mixture rye/oats lowered the MUFA level in comparison to the group fed with hybrid rye (*p* < 0.01). It was a very important observation because the anti-carcinogenic effect of these acids is well known [2,4,19].

In the previously published articles, the authors measured various MUFA levels in geese and chicken broilers lipids. In the Czech Republic geese, an approximately 42% MUFA level was reported [21]; in the Polish Kołudzka geese fed intensively, it was approximately 65% [22], and fed with corn silage, it was approximately 58% [23]; in the Ukrainian geese fed with a cereal mixture with soy oil, the MUFA level was approximately 29% [24]. The research on geese fed with rye proved that the MUFA level on average was 50.17%, which may be regarded as a desirable level. A similar level was obtained in chicken broilers fed with rye (approximately 51%). In chicken broilers fed with a commercial mixture based on soy flour, the MUFA level was approx. 41%, and for a mixture based on corn it was approx. 35% [25]; this qualified this product as less desirable due to health reasons. It is worth noticing is that the feeding of geese and chicken broilers with hybrid rye caused an increase in the cis configuration oleic acid C18:1 in the breast muscles. The positive effect of consuming monounsaturated fatty acids, especially the C18:1 acid, in atherosclerosis prevention was proven [19]. Such research was conducted after finding low atherosclerosis morbidity in those Mediterranean Sea countries where olive oil consumption, rich in oleic acid but poor in saturated acids, is common.

A significantly lower PUFA level was observed in geese fed with hybrid rye (about 19%) than in the oat group (about 22.5%), i.e., 3.4 pp less. A proportionally lower effect was observed in geese fed with a rye-oat mixture (approximately 1 pp lower).

Confirmed differences in PUFA n-6 and n-3 levels caused that the n-6/n-3 ratio was more profitable in the case of hybrid rye feeding of all the experimental birds. In chicken broilers meat, this ratio decreased with a higher rye dose, starting from 5.8 in the control group to 5.2 with 10% rye in the diet and to 4.6 in the group with 20% rye in the diet. In the meat of geese fed with oats, this ratio was 17.1, whereas in geese fed with rye 13.5 and geese fed with a rye-oat mixture, it was 13.2. A less positive n-6/n-3 ratio than obtained within this research was reported by Disetihe et al. (2019), in the experiment on chicken broilers feeding with a commercial mixture with soy (12.56) and with corn (8.99). A better n-6/n-3 ratio was reported by Uhlirova et al. [21], i.e., 9.04 to 10.28, depending on the experimental group of geese and in the research on feeding geese with soy oil i.e., 3.89 [24].

In generally, the positive effect was observed in fatty acids profile of geese fed only with hybrid rye or with the mixture hybrid rye and oats in proportion 1:1. However, diets with only hybrid rye caused smaller final body weight. For this reason, the diet with the above mixture is better for production targets and geese meat quality. Lower than in oats feeding, the final body weight of chicken broilers at 17^en^ week of age was stated in Karwowska et al. [23]’s study, which was, on average: 4850 g ± 507. In this study, geese were fed with beet pulp silage.

There was no effect of hybrid rye feeding on the total cholesterol level in geese muscles. In chicken broilers, all hybrid rye levels in the diet caused a significant decrease of cholesterol level in the breast muscle, from approximately 73 mg/100 g in the control group to approximately 60–65 mg/100 g in groups fed with 10% and 20% of hybrid rye in the diet. It is commonly known that cholesterol is present in all animal cell membranes. It is the simplest place for cholesterol to form, and that is why its amount is not correlated with fat content in meat. Chicken and turkey meat contains on average 65–73 mg/100 g of cholesterol [26,27], i.e., a level similar to the level obtained within this research.

Regardless of the above-established dependencies between the supplementation of hybrid rye in the diet of broilers and the fatty acid profile in meat, there are some changes in its qualitative characteristics. In their study, Lisiak et al. [28] determined with analogous food groups of geese that meat had some better physical and chemical traits, for instance lower fat content, lower drip loss, higher protein content, and better sensory quality in comparison to feeding with oats.

On the other hand, in our studies on the effect of hybrid rye in diet of the chicken broilers on the quality characteristics of meat, it was shown that its addition resulted in greater final acidification of the breast muscle, improvement in WHC, and lowering the b* parameter level. There was no effect of feeding with this rye on the sensory traits of the chicken meat.

## 5. Conclusions

No effect of feeding hybrid rye to geese on the SFA level was confirmed. However, the addition of 10% and 20% of hybrid rye in the diet lowered the SFA level in chicken broiler meat. The MUFA level was significantly dependent on the rye diet in geese and chicken broilers. Moreover, the MUFA level was higher and more positive when the addition of hybrid rye was higher. A significantly lower PUFA level in the meat of birds fed with hybrid rye was observed in geese. The n-6/n-3 PUFA ratio was significantly lower in all experimental birds fed, with the addition of hybrid rye which confirmed the benefits of including hybrid rye in the diets of these animals. The diet with mixture hybrid rye and oats in proportion 1:1 is better for production target and geese meat quality, because it was stated as having a better fatty acids profile and does not negatively effect the growth of geese. Generally, the feeding of geese and chicken broilers by the addition hybrid rye in the diet caused good quality of the meat, and in some traits, also led to an improvement in the comparison to the control group.

## Figures and Tables

**Table 1:** The experimental plan of the Zatorska geese breed fed with different kinds of cereals.

Weeks of Being Reared	Sample Size, *n*	Feeding Method
1 to 3	300 nestlings	Feed and water ad libitum. Composition of the concentrate and its nutritive value is shown in Table 2.
4 to 14	300 birds	Feed and water ad libitum. Access in pasture for at least 8 h per day. Composition of the concentrate and its nutritive value is shown in Table 2. At the end of 14th week, the birds were divided into 3 feed groups of males and 3 groups of females.
15 to 17	Group A *n* = 100	Fed only with hybrid rye Brasetto cultivare. Birds fed ad libitum with access to straw aviary.
	Group B *n* = 100	Fed with oats. Birds fed ad libitum with access to straw aviary.
	Group C *n* = 100	Concentrate of oats and hybrid rye Brasetto cultivare (1:1, by weight). Birds fed ad libitum with access to straw aviary.

**Table 2:** Composition of concentrates and chemical composition of feed used in geese feeding.

Composition, %	0–3 Weeks (Feed No. 1)	4–14 Weeks (Feed No. 2)
Feed phosphate	1.0	1.0
Limestone	1.8	1.2
Maize	40.0	35.0
Premix	1.2	1.1
Wheat	27.5	24.2
Wheat brains	0.00	10.0
Soya bean meal	28.5	23.5
Sunflower extracted meal	0.00	4.0
Crude protein	19.5	19.2
Crude fiber	2.8	3.8
Vegetable oils and crude fat	2.5	2.6
Crude ash	5.4	5.1
Lisine	0.97	0.91
Methionine	0.48	0.40
Calcium	0.94	0.73
Sodium	0.17	0.17
Available phosphorus	0.36	0.44
Metabolic energy. MJ/kg feed	11.50	10.20

**Table 3:** Chemical composition of hybrid rye and oats used for geese feeding after 98 days of life.

Composition, %	Hybrid Rye	Oats
Dry matter	87.87	89.41
Ash	1.58	3.23
Total protein	9.75	10.55
Crude fat	1.50	4.90
Crude fiber	1.94	13.21
Gross energy, kcal·kg^−1^ d.m.	4375	4534

**Table 4:** Composition and nutrient content of experimental diets for chicken broilers fed different doses of hybrid rye in diet (g/kg dry matter).

Specification	Starter (1–21 days)			Grower-Finisher (22–42 days)		
	0%	10%	20%	0%	10%	20%
Rye	0	100	300	0	100	300
Corn	457.1	354.1	302.1	404.4	298.4	238.4
Wheat	100	100	40	200	200	150
Soybean meal	370	365	370	306	304	307
Rapeseed oil	33	41	48	52	60	67
Monocalcium phosp.	15	15	15	13	13	13
Methionine	2.6	2.6	2.6	2.3	2.3	2.3
Metabolic energy, MJ/kg feed	12.6			13.1		
Crude protein	225			205		
Lysine	12.3			11.5		
Methionine	5.8			5.25		
Threonine	8.5			8.1		
Calcium	9.7			9.3		
Total phosphorus	7.1			6.6		

**Table 5:** Final body weight and fatty acids content in the breast muscles of geese fed with various cereals (%).

Specification	Type of Cereal Diet			SEM	*p* Value
	Rye, Group A	Oat, Group B	Rye/Oat, Group C		
Final body weight, g	4767 B	5148 A	4944	560	0.000
C 12:0	0.089 C	0.086 C	0.041 AB	0.004	0.000
C 14:0	0.353	0.373	0.393	0.008	0.131
C 14:1	0.003 B	0.033 AC	0.002 B	0.003	0.000
C 15:0	0.053 c	0.072	0.073 a	0.003	0.028
C 16:0	21.415	21.385	21.956	0.129	0.124
C 16:1	3.018	2.817	2.985	0.036	0.053
C 17:0	0.128	0.125	0.135	0.003	0.301
C 17:1	0.091 Bc	0.063 A	0.071 a	0.003	0.001
C 18:0	8.540 b	9.138 a	8.676	0.090	0.015
C 18:1 trans	0.324 BC	0.269 A	0.271 A	0.007	0.001
C 18:1 cis 9	43.123 BC	39.595 AC	41.423 AB	0.234	0.000
C 18:1 cis 11	2.880 b	2.724 ac	2.868 b	0.028	0.038
C 18:2 n-6	12.859 BC	15.500 AC	13.975 AB	0.175	0.000
C 18:3 g n-6	0.065	0.063	0.058	0.002	0.393
C 18:3 n-3	0.805 B	0.684 Ac	0.755 b	0.013	0.000
C 20:0	0.107	0.100	0.092	0.003	0.073
C 20:1	0.527	0.503	0.492	0.007	0.135
C 20:2 n -6	0.146 BC	0.185 A	0.173 A	0.004	0.000
C 20:3 n-6	0.145 B	0.184 AC	0.153 B	0.004	0.000
C 20:4 n-6	3.944 B	4.623 AC	3.916 B	0.087	0.001
C 20:5 n-3	0.085	0.068	0.073	0.006	0.568
C 22:4 n-6	0.596 B	0.708 AC	0.598 B	0.014	0.001
C 22:5 n-3	0.227	0.231	0.231	0.006	0.955
C 22:6 n-3	0215 BC	0.274 AC	0.378 AB	0.011	0.000
Cholesterol (mg/100 g)	78.913	77.325	75.300	0.713	0.116
SFA	30.686	31.310	31.368	0.159	0.153
MUFA	50.168 BC	46.137 AC	48.278 AB	0.269	0.000
PUFA	19.087 Bc	22.520 AC	20.310 aB	0.260	0.000
n-3	1.322	1.258 C	1.437 B	0.021	0.001
n-6	17.755 B	21.263 AC	18.873 B	0.257	0.000
n-6/n-3	13.478 B	17.064 AC	13.233 B	0.279	0.000

Means marked by the different lower case are significant at *p* < 0.05; Means marked by different uppercase are significant at *p* < 0.01.

**Table 6:** Final body weight, dressing yield and fatty acids content in the breast muscles of chicken broilers fed with different doses of hybrid rye (%).

Fatty Acids	Dose of Hybrid Rye in Diet			SEM	*p* Value
	0%, Group A	10%, Group B	20%, Group C		
Final body weight, g	3015	2988	2984	160	0.893
Dressing yield, %	79.30	80.09	79.78	1.94	0.678
C 12:0	0.103 bC	0.053 a	0.044 A	0.007	0.001
C 14:0	0.532 bC	0.469 aC	0.366 AB	0.013	0.000
C 14:1	0.076 C	0.078 C	0.059 AB	0.002	0.000
C 15:0	0.075 c	0.070	0.064 a	0.002	0.026
C 16:0	18.176 C	17.803 C	16.051 AB	0.151	0.000
C 16:1	3.565 C	3.589 C	3.021 AB	0.046	0.000
C 17:0	0.143 C	0.130	0.121 A	0.003	0.005
C 17:1	0.103 c	0.108	0.072 aB	0.005	0.005
C 18:0	6.820 cb	6.364 a	6.371 a	0.085	0.042
C 18:1 trans	0.185 C	0.190 C	0.12 AB	0.010	0.009
C 18:1 cis 9	41.148 BC	43.248 A	43.530 A	0.175	0.000
C 18:1 cis 11	2.760 BC	3.240 A	3.309 A	0.045	0.000
C 18:2 trans	0.032 C	0.035 c	0.065 Ab	0.005	0.005
C 18:2 n-6	18.457 B	17.167 Ac	18.048 b	0.158	0.002
C 18:3 g n-6	0.164	0.150	0.149	0.003	0.165
C 18:3 trans	0.076	0.125	0.083	0.009	0.051
C 18:3 n-3	2.788 C	2.828 C	3.408 AB	0.047	0.000
C 20:0	0.115	0.109	0.105	0.002	0.168
C 20:1	0.703 b	0.744 ac	0.704 b	0.008	0.039
C 20:2 n-6	0.280 B	0.248 A	0.258	0.004	0.005
C 20:3 n-6	0.291	0.275	0.293	0.007	0.547
C 20:4 n-6	1.653 c	1.462 C	1.932 aB	0.047	0.000
C 20:5 n-3	0.152 C	0.157 c	0.184 Ab	0.005	0.006
C 22:4 n-6	0.399 B	0.321 AC	0.392 B	0.010	0.001
C 22:5 n-3	0.503 C	0.490 C	0.646 AB	0.015	0.000
C 22:6 n-3	0.172 C	0.233	0.285 A	0.012	0.001
Cholesterol (mg/100 g)	72.500 BC	59.933 Ac	64.533 Ab	0.955	0.000
SFA	25.963 C	24.997 C	23.121 AB	0.228	0.000
MUFA	48.721 BC	51.326 A	50.914 A	0.196	0.000
PUFA	25.028 b	23.563 aC	25.813 B	0.235	0.000
n-3	3.615 C	3.707 C	4.523 A B	0.055	0.000
n-6	20.592 B	19.348 AC	20.780 B	0.160	0.000
n-6/n-3	5.807 BC	5.238 AC	4.598 AB	0.030	0.000

Means marked by different lower case are significant at *p* < 0.05; Means marked by different uppercase are significant at *p* < 0.01.

**Table 7:** Selected quality traits of breast muscles of chicken broilers fed with different amounts of cereals in diet.

Quality Traits	Dose Hybrid Rye in Diet			SEM	*p* Value
	0%, Group A	10%, Group B	20%, Group C		
pH_15′_	6.58	6.61	6.61	0.141	0.783
pH_24 h_	6.12 b	6.00 a	6.05	0.109	0.028
Color L*	52.52	52.02	52.89	2.564	0.501
Redness a*	2.91	1.99	2.07	1.512	0.066
Yellowness b*	0.63 BC	−0.63 A	−1.32 A	1.667	0.001
WHC, %	28.09 BC	21.84 A	22.12 A	4.639	0.001
Smell, points	4.47	4.55	4.53	0.192	0.340
Flavor, points	4.48	4.47	4.47	0.235	0.989
Juiciness, points	4.53	4.40	4.44	0.273	0.243
Tenderness, points	4.59	4.61	4.56	0.220	0.693

Where: L* means lightness, a* redness, b* yellowness and means marked by the different lower case are significant at *p* < 0.05; Means marked by different uppercase are significant at *p* < 0.01.

## Data Availability

Not applicable.
